# Development of Flexible and Functional Sequins Using Subtractive Technology and 3D Printing for Embroidered Wearable Textile Applications

**DOI:** 10.3390/ma14102633

**Published:** 2021-05-18

**Authors:** Ramona Nolden, Kerstin Zöll, Anne Schwarz-Pfeiffer

**Affiliations:** Research Institute for Textile and Clothing, Hochschule Niederrhein-University of Applied Sciences, Webschulstraße 31, 41065 Mönchengladbach, Germany; Kerstin.zoell@hs-niederrhein.de (K.Z.); anne.schwarz-pfeiffer@hs-niederrhein.de (A.S.-P.)

**Keywords:** 3D printing, additive manufacturing, circuit boards, functional sequins, subtractive technology, wearable electronics

## Abstract

Embroidery is often the preferred technology when rigid circuit boards need to be connected to sensors and electrodes by data transmission lines and integrated into textiles. Moreover, conventional circuit boards, like Lilypad Arduino, commonly lack softness and flexibility. One approach to overcome this drawback can be flexible sequins as a substrate carrier for circuit boards. In this paper, such an approach of the development of flexible and functional sequins and circuit boards for wearable textile applications using subtractive and additive technology is demonstrated. Applying these techniques, one-sided sequins and circuit boards are produced using wax printing and etching copper-clad foils, as well as using dual 3D printing of conventional isolating and electrically conductive materials. The resulting flexible and functional sequins are equipped with surface mounted devices, applied to textiles by an automated embroidery process and contacted with a conductive embroidery thread.

## 1. Introduction

Researching and developing the integration of new skills in materials science makes materials increasingly ‘intelligent’ and gives them new functions or sensory components. Especially in the field of textile technologies and wearable textile applications, the need for high design flexibility and customer-specific functionality is growing [1]. Furthermore, there is currently a great need for ever increasing conductor density, more complex multi-level circuits with simultaneous flexibilization of circuit boards and miniaturization of components.

The integration of electronic components with textiles for wearable textile applications is summarized under the term smart textiles. These textiles feel stimuli of the environment and react and adapt to them by integrating functionality into textile structures [2]. The combination of electronics and textiles is divided into three categories. The first category comprises textiles that serve as carriers for standard electronic components. Textiles, that are included in category two, are carriers, sensors and/or actuators and sometimes replace electronic components. Category three includes textiles that completely replace electronic components. The categorization creates a distinction between intelligent textiles with integrated electronic components and textiles that avoid electronics and act as such themselves [3].

There are already numerous first attempts of textile-integrated electronics up to marketable products, e.g., the iconic Levi’s^®^ trucker jacket, which combines style with innovative and smart jacquard technology [4]. Other examples are intelligent heatable underwear [5] or luminous ceiling tiles, that improve acoustic performance and ease the technical application in lighting installations [6]. Thereby, the integration technology of choice for these attempts is embroidery.

**Figure 1:**
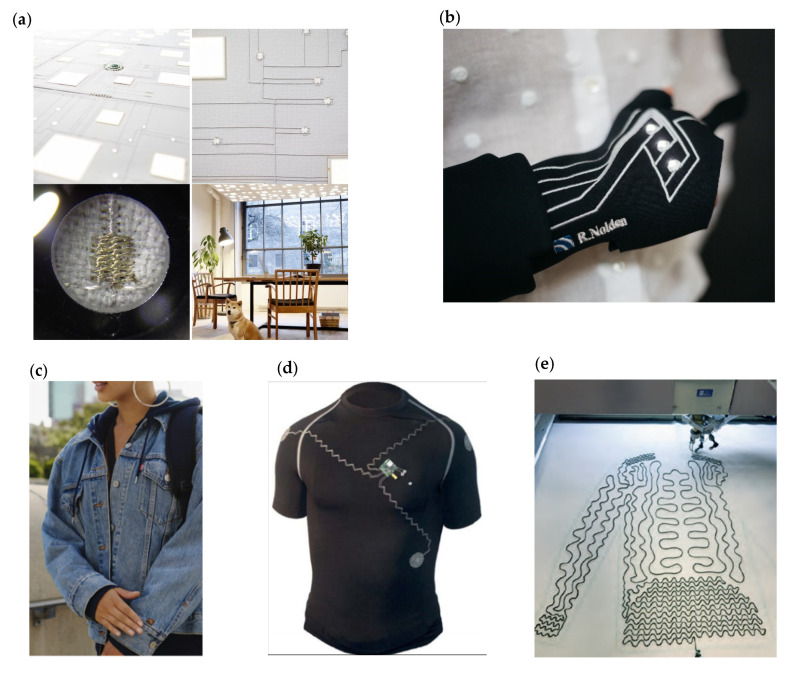
(**a**) Embroidered light for acoustic ceilings [6] (**b**) Smart glove [8] (**c**) Jacquard Trucker Jacket Levi’s^®^ [4] (**d**) EKG shirt with embroidered electrical interconnections [7] (**e**) Embroidered heating jacket [11].

Embroidery is a promising technology for the production of textile-integrated electronics and is already being used in various application fields. The combination of textile and electronics is applied for, e.g., bio-potential measurements as an electrocardiogram (EKG) in shirts. Thereby, textile electrodes are embroidered by moss technology and used to measure body functions [7]. Another application field is the healthcare sector lies in therapeutic and diagnostic devices. A smart glove that supports dedicated finger training is used for the rehabilitation of finger stiffness and arthritis. The bending sensor in the finger region and the signal visualization by functional sequins equipped with LEDs on the back of the hand, are realized by an automated embroidery technology [8]. Furthermore, a textile-integrated sensor-system, a knee bandage, is currently being developed to diagnose harmful knee movements with feedback-supported rehabilitation after surgery of the anterior cruciate ligament. Hereby, the data conductors and the electronic components are applied also by embroidery technology [9]. In addition, the application of conductive threads, metal wires, laminated polymer and carbon fibers by the Tailored Fibre Placement (TFP) process is also an important technology for the production of three-dimensional structures and e-textiles [10] (Figure 1).

In all presented applications, the sensors and data conductors are realized by embroidery with conductive embroidery yarns or wires. The evaluation unit for data storage, however, consists of rigid circuit boards such as Lilypad Arduino, Raspberry Pi or Sparkfun electronics [4,7,8].

Mechanical stress due to sharp edges of rigid circuit boards causes high abrasion of conductive yarns and recurring yarn breaks. This drawback obstructs the realization of textile-integrated electronics and textile wearables [12]. Therefore, circuit boards must become more flexible. In addition, the rigid circuit boards cannot automatically be applied during the embroidery process until now. With functional sequins, this drawback can be overcome as an automated application by an integrated sequin device on multifunctional embroidery machines.

The world-famous textile sequins are a millennium-old, exclusive piece of jewelry that the ancient Egyptians used to decorate their clothes in form of small gold plates. The foundation stone for the industrial production of sequin fabrics on Schiffli embroidery machines was laid by the St. Gallen textile company Jakob Schlaepfer with a world patent in 1963 with the start of industrial sequin embroidery in 1965 [13]. After a long success story in the textile fashion world, conventional textile sequins are no longer only used as decorative jewelry, but are increasingly being used as functional supports for electronic components, so-called functional sequin devices (FSDs).

The Research Institute Thüringen Vogtland e.V. has carried out a targeted further development of sequin technology, the so-called Functional Sequin Devices (FSDs), that is now distributed by Imbut GmbH (Greiz, Germany) [14]. Usual jewelry sequins are equipped with electronic components using automated assembly. For this purpose, a plastic carrier is coated with special conductive substrates from electrical engineering and provided with a corresponding circuit layout for different components of type 0805. This component shape corresponds to the size of the surface mounted devices, also called SMD design. Surface mounted device refers to surface mountable components. Due to their small size, these small components can be soldered directly onto the sequins without drilling beforehand. After substituting the circuit layout, the plastic carriers are cut into sequin ribbons. Following on from this process, the sequins are equipped with the electronic components.

The functional sequins can be placed on a textile in an automated process using embroidery technology, attached with conventional embroidery thread and contacted with a conductive embroidery thread. Not only is the contact made with the conductive embroidery thread; a complex embroidered circuit can also be realized. With the technology of functional sequins, standard processes of electrical engineering are combined with textile technologies and offer a very large research potential in the flexibilization of electronic carriers and components. The history of sequins and the development of functional sequins creates the possibility to manufacture flexible carrier structures instead of using of rigid circuit boards. Additionally, functionalization of textiles by an automated application of textile-integrated electronics and simultaneously maintaining the desired textile character becomes possible. This approach circumvents both the bottleneck of rigidity and thickness of circuit boards.

Currently, the functional sequins are produced by subtractive technology [14]. With the subtractive method, the carrier substrate is laminated on the surface, on one or both sides, with a full surface and holding copper foil. The conductor structures are created by etching away excess copper [15].

**Figure 2:**
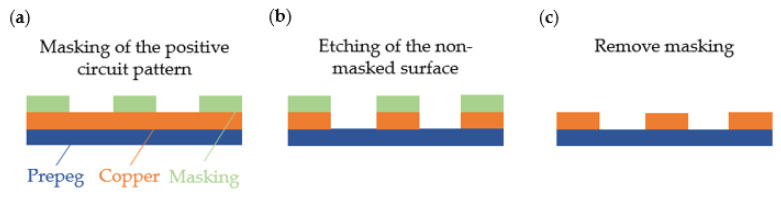
Subtractive technology (**a**) masking of the positive circuit pattern (**b**) etching of the non-masked surface (**c**) removing masking.

The positive conductor pattern of the electronically conductive structures is applied to the copper-coated circuit board using screen printing, photo or Citrak processes (Figure 2). An etch-resistant chemical is applied, which prevents the etching of printed areas in the later etching process. The most common and most advantageous etching method for etching copper is in acid solution with sodium persulfate or iron (III) chloride, as etched copper in the solution can easily be removed by electrolysis or precipitation. In order to prevent the formation of oxide layers on the copper surface, it is coated with a resistant metal alloy made of, e.g., tin silver or nickel and additionally a solder resist and protective mask can be applied [16].

**Figure 3:**
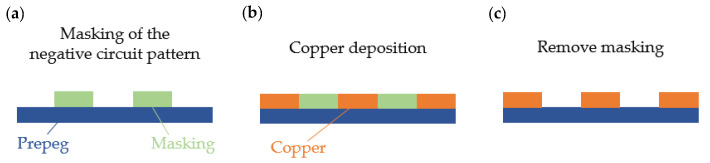
Additive technology (**a**) masking of the negative circuit pattern (**b**) copper deposition (**c**) removing masking.

In contrast to the subtractive method, the additive technology does not require the copper to be applied over the entire surface but only where it is needed as a conductive connection [15]. Therefore, the additive method is more sustainable and resource-efficient. By printing a negative conductor pattern using screen printing or photo printing, the areas that should not be electrically conductive are masked (Figure 3).

The additive structure of the conductor pattern is created by electroless chemical copper plating, thin film technology or thick film technology [15]. The after treatment is similar to the subtractive technique. The mask print is removed by cleaning and the conductive copper surfaces are refined and provided with solder resist and protective lacquers.

3D printing can also be categorized as additive technology, that is a generative and additive manufacturing process in which models, samples, prototypes, tools and end products can be manufactured quickly and inexpensively [17]. One process of 3D printing is the fused deposition modeling (FDM) or fused filament fabrication (FFF), where a meltable thermoplastic and wire-shaped plastic or wax material is tempered to the melting point in an extruder and then pressed in layers from the movable and heated extruder nozzle. Common materials are acrylonitrile butadiene styrene (ABS), polylactide (PLA), polyvinyl alcohol (PVA) and thermoplastic polyurethane (TPU) [18]. In addition to the known polymers, there are electrically conductive plastics that consist of a thermoplastic polymer that is mixed with conductive particles such as carbon black (proto-pasta [19]) or copper (Electrifi from Multi3D [20]).

In this paper, the subtractive and additive techniques for the development of flexible and functional sequins are tested and compared. The substrates need to become thinner and more flexible as to minimize thread breakage when contacting conductive yarn in the embroidery process. Ultimately, the functionality of the developed functional sequins is proven by equipping them with LEDs.

The newest requirements of circuit boards for textile-integrated electronics and wearable textile applications can be implemented using flexible printed electronics. Particularly, the conventional electrical engineering offers numerous solutions with high innovational potential.

Flexible printed electronics have special features that bring many new and unexpected applications to our daily lives, such as interactive displays, flexible sensors and displays and data processing circuits that can be adapted to 3D objects, human skin or textiles. Due to the low production costs of those printing processes, these electronic products can be manufactured more cost-effectively and flexible than comparable other types of electronics [21].

**Figure 4:**
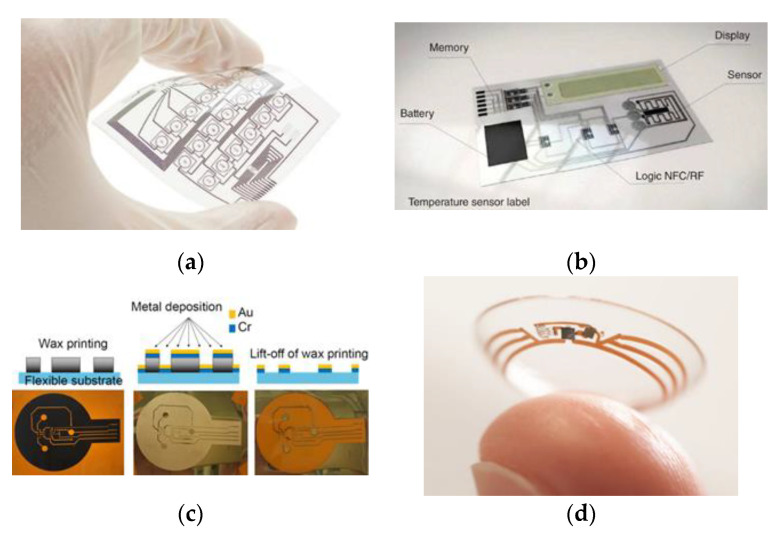
(**a**) Printed flexible circuit board [22] (**b**) Printed temperature sensor using inkjet printer [21] (**c**) Subtractive printing [23] (**d**) Intelligent contact lens using inkjet printing [25].

Quad Industries [22] is already presenting first approaches to printed textile and flexible printed circuit boards and sensors. They currently offer two types of technology: printed sensors and flexible heating. The special printing technology from Quad Industries (St. Niklaas, Belgium) enables the sensors printing on wafer-thin, flexible and elastic foils or directly on textiles. Qui [23] demonstrates in his research work the printing of the negative conductor pattern with wax on a pre-treated Kapton^®^ film (polyimide film) with subsequent metallization of the conductor structures using sputtering. Thereby, a first sputtered layer of the conductor structure is based on chrome in order to ensure a fixation between the Kapton^®^ film (DuPont^TM^, Neu-Isenburg/Wilmington, NC, USA) and the subsequent silver layer to be sputtered. After sputtering, the wax layer is washed off with boiling water. The corresponding electrical components are applied with a silver-containing adhesive and insulated with a polydimethylsiloxane layer (PDMS). Cui [21] describes the printing of flexible circuit boards using an inkjet printer. A circuit board with integrated temperature sensors, printed battery patches and an electrochromic display for the formation of flexible sensor tags is demonstrated. The conductor structures are printed on a polyimide film with a silver-based and a carbon-based ink. A new method of developing flexible electronics is hybrid printing, presented by Valentine et al. [24]. Conductive and dielectric elastomeric materials are printed using an ink printer and are combined with the automated assembly of surface-mounted electronic components within a single integrated additive manufacturing platform. In this process, the insulating matrix is either printed with sensory conductor structures or equipped with electronic components with subsequent printing of the electrical connections (Figure 4).

Vuorinen et al. [26] have developed a printed temperature sensor consisting of a multilayer plaster structure. A protective paper and an adhesive layer are laminated on a polyethylene film. A polyurethane layer is applied to the adhesive layer, on the surface a meandering structure of conductive graphene PEDOT: PSS is printed with the inkjet print, which functions as the temperature sensor. Finally, silver conductors are applied using screen printing. Google and the pharmaceutical company Novartis (Basel, Switzerland) [25] present a special variant of a printed and miniaturized sensor using an ink printer. The ‘intelligent’ contact lens is used for non-invasive blood sugar measurement for diabetics by detecting the tear fluid. The developed smart lens technology consists of a lens made of conventional lens hydrogel material with an integrated wireless SMD chip, which contains a miniaturized glucose sensor and a battery, which is embedded between two layers. A hole in the lens enables the tear fluid to enter the sensor, which then analyzes the blood sugar levels.

All these publications [21,22,23,24,25,26] mainly present flexible solutions for the production of printed circuit boards using an inkjet printer. The conventional approach of, e.g., masking and etching surface-coated printed circuit boards using subtractive technology [15] continues to offer an innovative and untapped research need for the development of new, flexible and functional boards and sequins. For example, subtractive technology with mask printing [23] and etching technology offers a conceivable approach for realizing printed flexible printed circuit boards.

**Figure 5:**
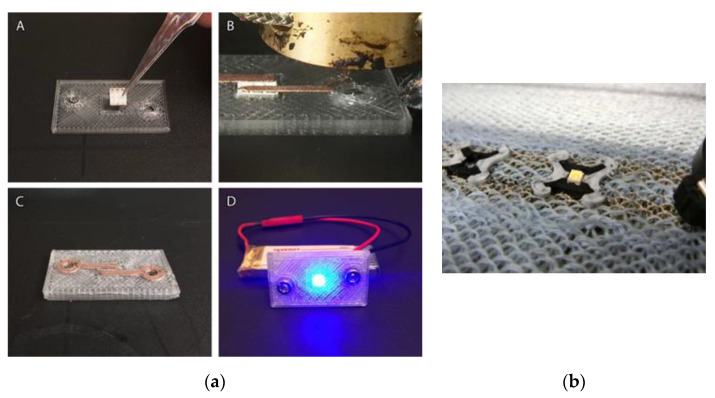
(**a**) Dual 3D printed circuit board (A) Inserting a LED component (B) Printing conductive traces (C) Embedded LED (D) Attached battery [30] (**b**) Dual 3D printing on textile fabric [29].

Wu et al. [27] works on 3D printed microelectronics such as resistors, capacitors and integrated wireless sensors. First, polymers and wax are printed. Subsequently, the wax is removed, which leaves hollow structures. Liquid metal is injected and hardened into these cavities. In a comparable research approach from Yanfeng et al. [28], a cruise control with direct printing technology and conductive inks is implemented using the molded interconnect device process (MID). The mechanical structure of a cruise control used in automobiles is printed by the injection molding process, leaving empty spaces for LEDs, plugs, resistors and electrical cables. The electrical connections and structures are printed with a silver ink and the resistors with a carbon nanotube (CNT) polymer composite. A solution for 3D printing directly on textiles is presented by the Bielefeld University of Applied Sciences, Department of Engineering and Mathematics [29]. A textile knitted fabric is made of a polyester and wool blend with conductor structures made of conductive wires made of uncoated copper and stainless steel and the conductive fiber yarn Shieldex^®^ (Statex). Fused Filament Fabrication (FFF) is applied to print the electrical connections between the textile and a LED using the conductive 3D printing filament Proto-Pasta. A contour made of PLA stabilizes the connection. The direct printing of the conductor structures and the supporting structure made of PLA on the textile is particularly advantageous, so that no further process for applying and fixing takes place (Figure 5).

There is further potential for miniaturization and increasing the flexibility of printing for this process. Flowers et al. [30] offers a solution of 3D printed circuit boards with conventional 3D printing filament as the carrier substance and conductive plastic as the conductor structure. The research project presents the comparison of different 3D printed conductors made of conductive plastics, the filaments Proto Pasta (Vancouver, WA, USA), Black Magic (Black Magic 3D, Ronkonkoma, NY, USA) and Electrifi (Multi3D, Cary, NC, USA). The Electrifi filament made of polyester with copper particles has the lowest electrical resistance in comparison to Proto Pasta and Black Magic, so a LED circuit board is printed with Fused Filament Fabrication Technology (FFF) by using the conductive Electrifi filament for conductive traces. The 3D printed carrier board is first printed out of PLA while then the printing process paused for assembly. The carrier board is removed from the 3D printer and the LED component is inserted into the corresponding recess. The 3D printing filament is changed at the same time and subsequently the printing is continued. Immediately during the printing process, the LED is contacted by the conductive filament on the contact surfaces. The conductor structures of the anode and cathode run in two holes, which are used to connect a current source using push buttons. The first approaches to 4D printing by Khoo et al. [31] are enabled by the combination of shape memory materials with conductive materials. A photopolymer or shape memory polymer is printed in a heated bath using the projection stereolithography (SL) process. The conductive ink is printed on the shape memory material using an inkjet printer. The shape memory polymer is heated by applying a voltage, the circuit is closed and an LED is illuminated.

3D printing offers a high research potential for the production of 3D printed electronic structures. 3D and 4D technology allow the production of robotics, sensors and portable electronics. However, previous research is subjected to three restrictions: process, material and design. If the restrictions can be overcome, an enormous change in the design and manufacturing systems of objects and devices would be possible [1]. The principle of additive manufacturing using dual 3D printing offers a highly innovative approach to the development of electronic circuit boards and functional sequins for wearables with textile-integrated electronics.

## 2. Applied Materials and Methods

### 2.1. Materials for the Subtractive Technology

For the subtractive technology two different base materials are used. The first base material is a conventional film made of polyethylene terephthalate (PET), which is coated with a copper adhesive tape from Tru Components (Las Vegas, NV, USA) of type CFT25/10M. It consists of a copper layer of 18 µm, which is provided with an acrylic adhesive with a layer thickness of 32 µm. The total material thickness is 50 µm. The second type is a copper-clad printed circuit board, which has a total material thickness of 50 µm. The one-sided copper coating is 35 µm thick. The plate material consists of a 15 µm thick epoxy glass fiber laminate.

**Figure 6:**
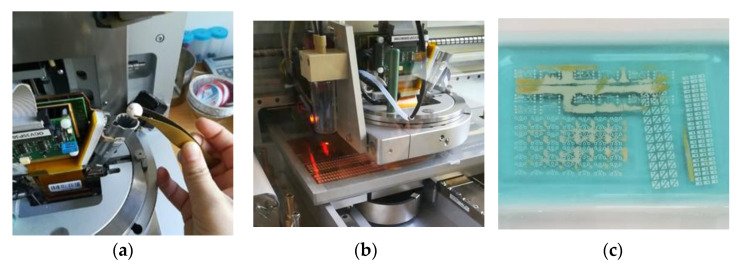
(**a**) Filling the wax and preparing the print of conductor pattern (**b**) Wax printing process (**c**) Etching process.

With the PixDRO LP50 wax printer from OTB Solar (Eindhoven, Netherlands) and Roth & Rau (Hohenstein-Ernstthal, Germany), a corresponding positive conductor pattern is printed on the respective copper-clad film carrier with wax (Figure 6). The wax beads are made of pure colorless wax and contain no additional chemical components. The intellectual hot melt print head for printing wax is a heated material supply for heating up to 70 °C. The print head has 256 nozzles, which print a minimum of one dot per inch.

The etching process takes place in a tempered sodium peroxodisulfate solution (Figure 6). The solution is heated to 50 °C with a temperature control plate. The temperature increases the rate of reaction of the etching effect. The samples are completely immersed in the solution for a uniform etching.

Two different chemicals are used to remove the wax. In the case of prints with PET film with copper adhesive tape, ethanol is used to prevent the waxy copper surfaces. The wax layer on the single-sided and double-sided copper-clad printed circuit boards is removed with acetone. In this case, there is no risk of detaching the copper lamination from the printed circuit board.

The sequin bands are cut with the single-ply cutter Zünd G3L-2500 from Zünd Systemtechnik AG (Altstätten, Switzerland) by using the drag knife universal cut tool (UCT). This is a knife that is pulled through the material to be cut without oscillating.

### 2.2. Materials for 3D Printing

With the aid of the Ultimaker 3 3D printer (Ultimaker, Utrecht, Netherlands), conventional 3D printing filaments made of Ultimaker 74526 PLA filament (color silver metallic), Ultimaker 74999 PVA filament M0952 (color natural), Ultimaker 74739 ABS filament M2560 (color gold) are used to produce the sequin base. Electrifi Conductive Filament from Multi3D (Cary, NC, USA) is used for the conductive structures of the printed sequins.

Sputtering of the 3D printed sequins is done with the Cressington Sputter Coater 108 auto (TESCAN GmbH, Dortmund, Germany). The target consists of a gold foil. Argon is used as the process gas. The gold is deposited on the sample with a duration of 60 s and a process pressure of 40 milliamps.

### 2.3. Electronic Components

The light emitting diode LTW-C191TS5 from Lite-On Technology Corporation (Taipei, Taiwan) is selected. These LEDs have the design 0603, which belong to the smallest housing types of SMD technology. The standardized dimensions of the LED are 1.60 mm in length and 0.80 mm in width, with a height of 0.55 mm. The insulating width between the cathode and the anode is 1.00 mm. The LEDs require a voltage of 5 volts (V) and a current of 20 milliamps (mA).

The RGB LED from Osram (Munich, Germany) of the LRTB GVTG type has a length of 3.5 mm and a width of 3.6 mm. The height is 2.0 mm. The required voltage of the RGB LED corresponds to 1.8 V to 3.7 V at a current of 50 mA.

A conductive adhesive with silver particles is used to populate the cut sequin ribbons with the different conductor pattern designs, which is dosed onto the conductive copper contact surfaces using a syringe and a fine needle attachment. The corresponding SMD components are then placed on the adhesive. The additional soldering of the contacts that have already been glued is omitted as to ensure the flexibility of the sequin tapes and not to stiffen with solder paste.

### 2.4. Materials for Application and Contacting during the Embroidery Process

A multifunctional portal-embroidery machine (type SGVA) with three embroidery heads, the F-, K- and W-head from ZSK Stickmaschinen GmbH, Krefeld, Germany, is used to process the conductive embroidery thread and the functional sequins. The transmission lines are produced by using the F-Head, which is a multi-needle head. It is based on a two-thread system and the stitch type is the double lockstitch. In order to achieve a low electrical resistance of the textile data transmission lines, three overlapping zig-zag double lockstitch seam lines are embroidered. The F-head is also applied to place and fix the functional sequins by a fully automated process.

The silver-coated polyamide embroidery thread, brand name HC 40 from Madeira Garnfabrik, Freiburg, Germany, serves as an electrical conductor and for contacting the sequins. It consists of 100% polyamide and is fully silvered and known by the name Shieldex^®^ from Statex (Bremen, Germany). The electrical resistance of the thread is less than 300 Ω/m. It can be machine washed at 30 °C and is certified according to Öko-Tex^®^ Standard 100 (Zürich, Switzerland).

## 3. Results and Discussion

The innovative approach to the development of flexible and functional sequins and circuit boards for wearable textile applications is subjected to the requirements of integrating embedded electronics and sensors, showing design and material-specific flexibility in a mini-status format and enabling the combination with textile technology. Previous designs of electronic components on rigid circuit boards influence the comfort and flexibility of textiles. More flexible, thinner and lighter circuit boards enable targeted integration of electronic components for wearable textile applications that are adapted to the textile requirements.

Considering the conventional manufacturing processes of electronic circuit boards, as well as 3D and wax printing, two modified processes are presented for the subtractive and additive technology, which are used to develop flexible and functional sequins and circuit boards. The functional sequins for subtractive technology and also additive manufacturing, are designed for single-sided pattern for two-pole LED surface mounted devices and multi-pole RGB SMD components. The single-sided conductor pattern for two-pole SMD components is designed for the application of SMD LEDs. The RGB LED is used for the design of a one-sided conductor pattern for multi-pole SMD components. The design is not created for a single sequin, but rather as a sequin band with sequins in a row. For contacting and applying the LEDs and RGBs, parallel and rectangular shapes and conductor traces are designed with a hole that represent the cathode and anode.

### 3.1. Subtractive Technology

The modified manufacturing concept of the subtractive technology includes the development of flexible and functional sequins made of copper-clad film carriers, which are reserved with wax printing with a positive conductor pattern and excess copper is removed by etching. Two manufacturing concepts with different film carriers serve this purpose.

**Figure 7:**
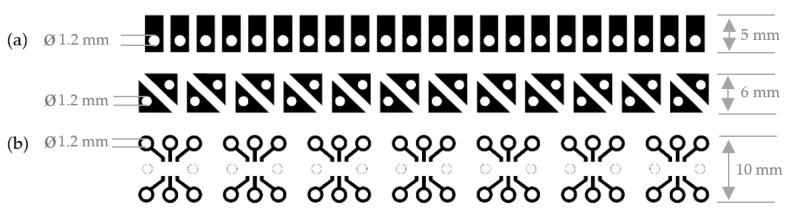
(**a**) Single-layer two-pole LED sequin tape designs and (**b**) multi-pole RGB sequin tape designs.

The first manufacturing concept uses a conventional film made of polyethylene terephthalate (PET), which is coated with a copper adhesive tape. For the second manufacturing process the copper-clad printed circuit board is used. The pattern is created in a CAD program and processed with the appropriate translation software of the wax printer. The single-layer designs for two-pole LEDs and multi-pole RGBs are shown in Figure 7.

After printing with the wax printer, etching of the unprinted and unreserved copper surfaces and the chemically cleaning of excess adhesive residues follows.

All samples are equally very flexible and foldable without cracking the wafer-thin copper layers. The surface on the copper-glued PET film is smooth and the copper surfaces are hardly noticeable because the layer the layer has a board thickness of just 0.14 mm. The surface of the copper-clad printed circuit board is matt and slightly roughened. The board is 0.05 mm thick.

**Figure 8:**
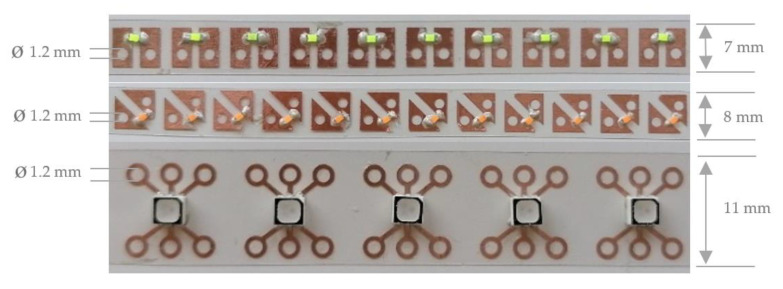
Printed single-layer LED and RGB sequin tapes on PET film with copper adhesive tape.

Finally, the printed sequins are cut to sequin ribbons suitable for the automated embroidery process and are equipped with the surface mounted devices. Figure 8 shows the printed single-layer LED and RGB sequin tapes on PET film with copper adhesive tape equipped with the electronic components.

**Figure 9:**
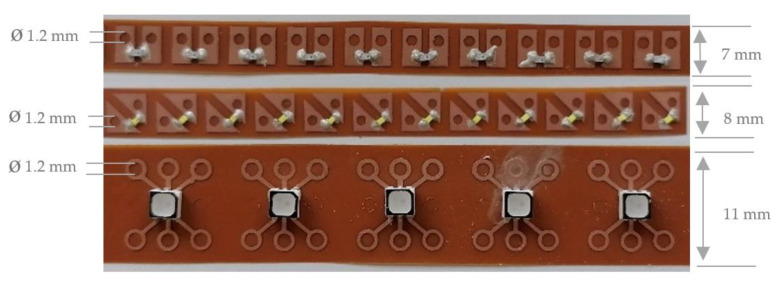
Printed single-layer LED and RGB sequin tapes on copper-clad film.

Figure 9 presents the printed single-layer LED and RGB sequin tapes on copper-clad film and as carrier of the surface mounted devices.

**Figure 10:**
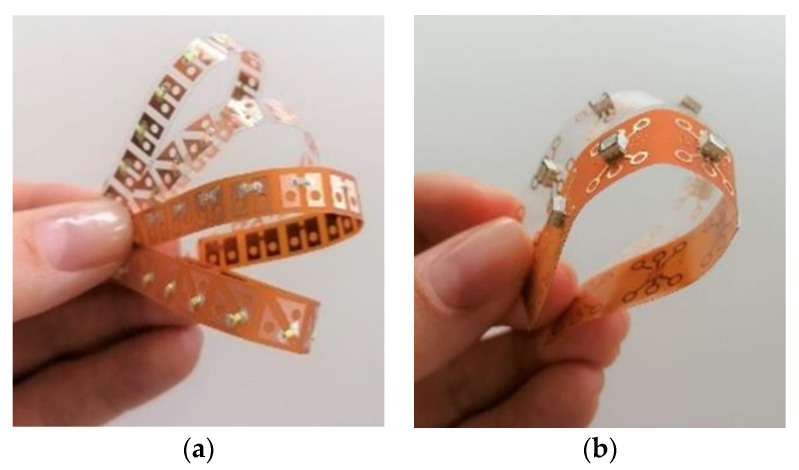
Printed sequin tapes: (**a**) LED sequin tapes and (**b**) RGB sequin tapes.

The sequin tapes are very flexible and the electronic components and the copper surface are stable against bending and twisting (Figure 10).

With the subtractive technology process of wax printing to mask a positive conductor pattern and subsequent etching process to remove excess copper, one-sided sequins tapes can be produced. The sequins from PET film with copper adhesive tape offers optically uniform and contour-fine conductor structures with a shiny and smooth copper film surface. The etching process has a shorter etching time than the etching time of copper-clad printed circuit boards samples. This is due to the production-related, untreated copper surface of the copper adhesive tape. The existing acrylic adhesive of the copper adhesive tape requires the etched samples to be freed from the wax masking but also from the acrylic adhesive residues. This additional cleaning is complex and slightly damages the conductor structures. It can lead to defects and peeling of the copper in places, especially of fine conductor lines and angular conductor areas.

The sequin ribbons made of copper-clad printed circuit board have a matt and rough surface. The etching process, on the other hand, takes longer for the samples made of PET film, since the copper surface is provided with a protective layer against oxidation. No glue residues need to be removed after the etching. The wax is removed quickly and does not damage the conductor structures. No defects are formed and the copper does not have to be detached. Only the contours of the conductor structures are slightly corrugated and not linear, since there is a slight undercut in the etching, which assault the contours. However, this has no impact on the result. Fitting with conductive silver adhesive does not affect the flexibility of the sequin tapes and boards.

### 3.2. Additive Manufacturing

Novel 3D printed functional sequins are manufactured using the dual 3D printing principle of fused filament fabrication (FFF). In one production step, two different polymers are automatically produced in any sequin and circuit board shape with varying conductor layouts for electronic components. The conductor structure can be covered in one layer on the surface or in multiple layers in the component, as well as printed on the surface. Thereby complex possibilities of circuit structures can be realized. The printing of flexible sequins and the integration of the conductor structures only in areas where it is needed, is based on the additive manufacturing process of the circuit board technology.

**Figure 11:**
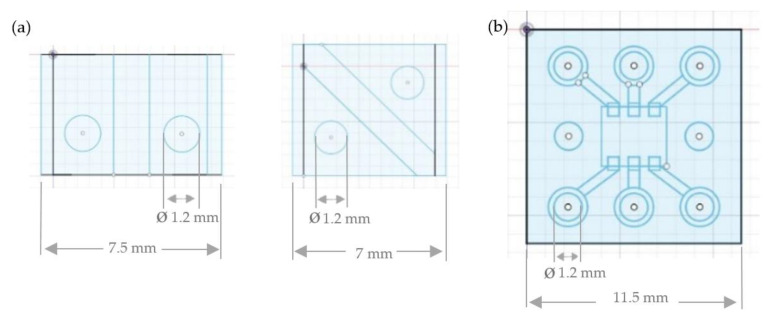
Single-layer sequin designs for (**a**) LED sequins designs and (**b**) RGB sequins.

The design construct of the functional sequin is created in the CAD software Autodesk^®^ Fusion 360^TM^ (Version 2.0.10032). The resulting volume shape is also sliced digitally in the open source 3D printer cutting software Ultimaker Cura (Version 4.8.0). In Figure 11 the single-layer LED sequin designs and the single-layer RGB sequin designs are visualized.

Three different 3D printing concepts are used to print the 3D sequins. The first concept is based on the 3D printing of a sequin made of PLA and the creation of the conductor structures using copper adhesive tape. In the second concept, the sequin carrier is printed from PLA. With the second nozzle of the dual 3D printer, a mask is printed on the sequin carrier with a water-soluble PVA filament. The unmasked areas are metallized with gold in a sputtering process. In the third concept, the sequin carrier is made of PLA and the conductor structures are printed with the conductive plastic filament Electrifi by using dual 3D printing.

#### 3.2.1. 3D Printing with PLA Filament and Gluing with Copper Adhesive Tape

**Figure 12:**
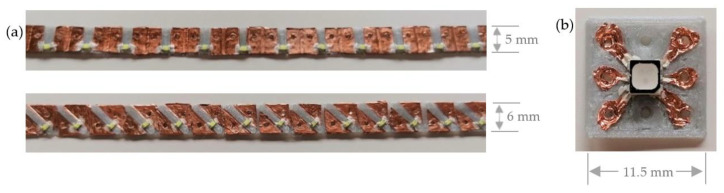
3D printed sequins with conductive copper foil structures: (**a**) PLA LED sequins and (**b**) PLA RGB sequin.

In the first concept, the 3D printed sequins are based on PLA. The one-sided sequins for the two-pole SMD LED components and the multi-pole RGBs are provided with a maximum material thickness of 0.6 mm by extrusion of the drawing. The areas that are coated with the conductive copper adhesive tape in a later step are given an extrusion height of 0.4 mm. This ensures that the PLA sequin achieves a uniform sequin thickness. On the right and left of the sequins, cut edges of 0.5 mm with a flattening 0.2 mm thick layer plane are created. With the help of the cut edges, several individual sequins can be joined to form a sequin band. The conductive structures are realized by using copper adhesive tape from Tru Components of type CFT25/10M, which is attached manually. The sequins glued with copper are equipped with the conductive adhesive with silver particles in order to create a flexible connection of the SMD LEDs with the sequins. In Figure 12 the realized LED and RGB sequins are shown.

The produced LED and RGB sequins are very thin and flexible due to the low material thickness. The advantage of this manufacturing process is the easy marking of the adhesive levels of the conductive copper surfaces by different levels of extrusion of the sequins. The cut edges of the LED sequin tapes can be easily separated from one another by bending or a vertical force, which is a prerequisite for the automated embroidery process. Equally positive is the short printing time of 3D printing of just a few minutes for a sequin band consisting of 15 sequins. However, the manual process step of gluing with copper tape is very complex. Automation is also not possible.

#### 3.2.2. 3D Printing with PLA and PVA Filaments and Metallization Using Gold Sputtering

**Figure 13:**
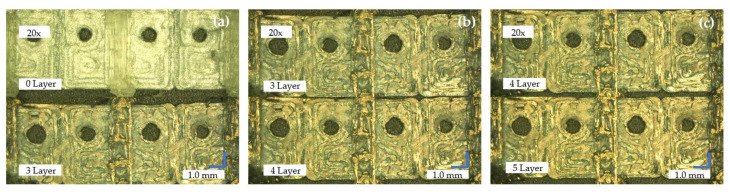
Microscopic images of the deposited gold layers. (**a**) PVA reservation without deposited gold layer at the top and the sequin ribbon with three coated gold layers at the bottom (**b**) three gold layers at the top and four gold layers at the bottom (**c**) four layers of gold particles on the top and five gold layers on the bottom.

The 3D printing of a negative model in the form of a sequin made of PLA filament and masking using water-soluble PVA filament is combined with the sputtering technique for metallizing the required conductor structures in the second concept. Five plating processes are carried out to deposit five layers of gold on the sequin belt. Figure 13 shows the 3D printed sequin tape (a) with PVA reservation at the top left and the sequin ribbon with three coated gold layers at the bottom. The comparison between (b) three gold layers at the top and four gold layers at the bottom is shown in the middle. The right micrograph shows (c) four layers of gold particles on the top and five on the bottom.

The optical layer thickness between the individual sputter processes is only slightly increased, which results in a time-consuming process with high target consumption. The maximally coated gold layers are measured with a multimeter and have no conductivity. Consequently, the five gold layers applied are not sufficient to deposit a suitable conductive coating on the 3D printed sequins. A large number of additional layers are required, however, due to the high target consumption, this approach is not further carried out. Considering the targeted productivity and automatization of the process though, sputtering with gold can be suggested for further experiments.

This concept, as well as the manufacturing concept of 3D printing of PLA sequins and gluing with copper adhesive tape, allows easy marking of the areas to be metallized by 3D modeling of the sequins. Cut edges for the production of sequin tapes are also integrated. However, the high process costs of sputtering and the numerous process steps required to produce the functional sequin are disadvantageous.

#### 3.2.3. 3D Printing with PLA and ABS Filament and Conductive 3D Filament

**Figure 14:**
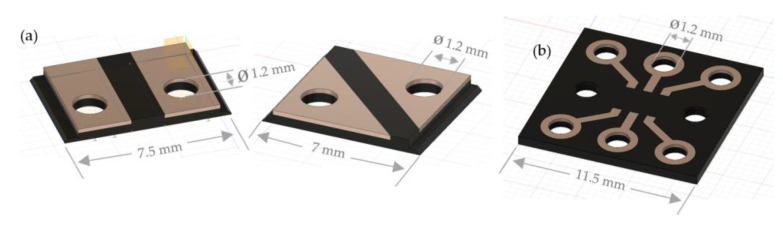
3D sequin designs for (**a**) LEDs and (**b**) RGBs.

Before the flexible and functional sequins are printed with conventional and conductive filaments based on the third concept, the designs are tested with the conventional 3D printing materials PLA and ABS with the print heads AA 0.4 and AA 0.6 from Ultimaker 3. This printing test is necessary to compare and print the sequin structures with the different print heads. The designs of the one-sided sequin for LEDs and the one-sided sequin for the SMD-RGB component are created in the Autodesk^®^ Fusion 360^TM^ software (Version 2.0.10032) (Figure 14) with the two material combinations of the PLA filament for the carrier board and the ABS filament for the conductor structures in a 3D shape by extrusion modeling.

The printing tests for the single-layer LED and RGB sequins can be printed evenly and without errors for the respective material and printing combinations mentioned in Table 1.

**Figure 15:**
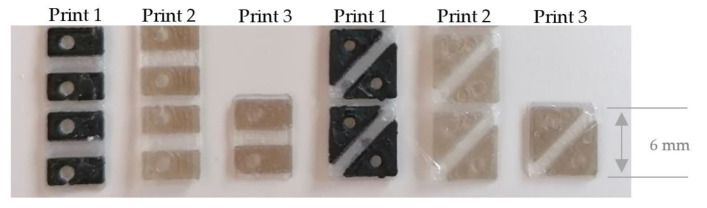
3D printed single-layer LED sequins and sequin tapes with different print cores and various 3D printing materials.

**Figure 16:**
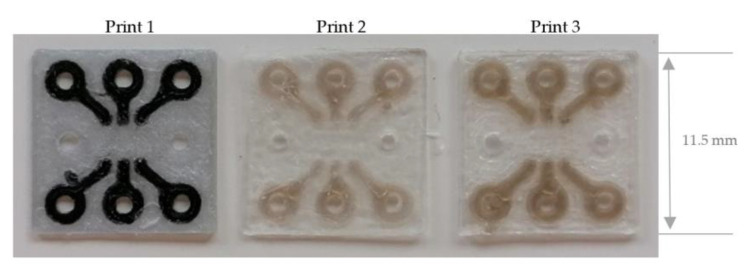
3D printed single-layer RGB sequins with different print cores and various 3D printing materials.

In Figure 15 and Figure 16 the sequins with PLA silver and PLA black, printed with the print head AA 0.4 (print 1), are shown on the left. The material combinations PLA transparent and ABS gold, printed with the print head AA 0.4 (print 2), are visualized in the middle. On the right, PLA printed with the print head AA 0.4 and ABS, printed with the print head AA 0.6 (print 3) are demonstrated.

The difference between the nozzle openings of the AA 0.4 and AA 0.6 print heads lies in the higher extrusion or filling volume of the conductor areas with the AA 0.6 print head. The structures are fuller with a simultaneously reduced diameter of the bores of the contacts, which means that the AA 0.4 print head is more suitable for printing the functional sequins and enables finer structures.

#### 3.2.4. 3D Printing with PLA and ABS Filaments and Conductive 3D Filament

The modeled 3D designs of the single-layer LED and RGB sequins for 3D printing with PLA and ABS filaments are used in the third concept. In the slice software Cura (Version 4.8.0), the parameters of the ABS filament are replaced by the slice parameters of the conductive Electrifi filament.

**Figure 17:**
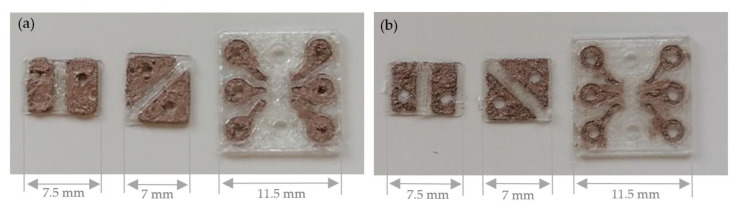
3D printed LED and RGB sequins at 95% material flow with the conductive filament Electrifi from Multi3D (**a**) print head AA 0.4 (**b**) print head BB 0.4.

Although the print tests with PLA and ABS filaments revealed that the print head AA 0.6 is not primarily suitable for fine conductor structures, the print tests of the functional sequins with the Electrifi filament are nevertheless carried out with the print heads AA 0.6 and BB 0.4 in order to compare the print properties. The standardized material flow of the 3D printer of 100% leads to an excessive material extrusion when printing the Electrifi filament. The conductive structures of the LED and RGB sequins are uneven for both print heads and run in the contours. Even when reducing the material flow to 95% for the Electrifi filament, the conductive structures remain uneven (Figure 17).

During the printing process it is observed that both print heads, AA 0.6 and BB 0.4, slightly wiped off the already extruded filament with the edge of the nozzle opening. It leads to an uneven distribution, continuous contours and an irregular and uneven printing result. Even if the material flow is reduced, the extruded material is captured by the nozzle and spread. On the one hand, this phenomenon is caused by the flat tip of the nozzle design, on the other hand, due to the viscosity or the thermal behavior of the extruded polyester filament. The PLA and ABS materials solidify quickly after printing. The Electrifi filament remains longer in the softened state due to the high printing temperature required, which makes it easy for the print head nozzle to catch it. However, if the printing temperature of the Electrifi filament is reduced in order to shorten the time for the printed filament to cool and solidify, the nozzle becomes blocked.

**Figure 18:**
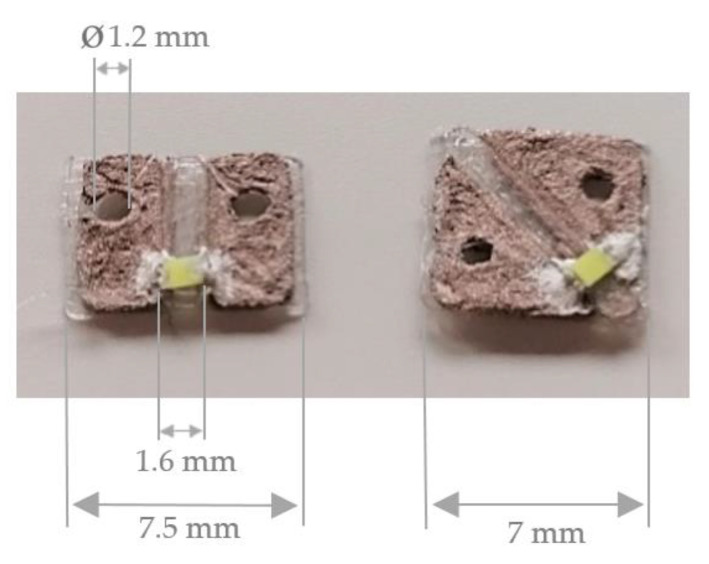
3D printed PLA LED sequins with conductive filament Electrifi from Multi3D and equipped with LEDs.

Only the LED sequins, printed with the BB 0.4 print head, can be equipped with SMD LED components (Figure 18), while the RGB sequins do not allow further processing as the conductive structures show non-uniformity of the print at some points. The silver adhesive sticks well to the Electrifi material. Through the non-uniformity and the roughness of the conductive surfaces make it difficult to attach the SMD LEDs, so these cannot be applied flat to the surface of the sequins.

Considering the printing tests carried out, functional 3D printed sequins cannot be produced productively with a conventional PLA filament and a conductive filament in a dual 3D printer. This theoretical manufacturing concept offers the production of thin, flat, flexible and functional sequins and blanks in just one process step, which unfortunately cannot be printed with a conventional 3D printer that is also suitable for home use.

According to the first concept with conventional PLA filament and the conductive structures with copper adhesive tape, very thin and flexible functional LED and RGB sequins, as well as sequin tapes can be produced. The construction as a sequin tape enables the integration of cut edges, which are required for automated application and contact using an embroidery machine. The concept convinces with a short printing time of the 3D sequin shape. Manual gluing of the conductive surfaces and structures, however, is time-consuming. In addition, it is not possible to automate the subsequent gluing of the sequins after 3D printing. The second concept combines the 3D printing of sequins from PLA with PVA masking and the metallization of the conductor structures using gold sputtering. The dual 3D printing of the sequins is automated within a few minutes. The combination of individual sequins to form a sequin band and the integration of cut edges are possible. Metallization by sputtering with gold particles, however, has a very high target consumption that, in combination with an insufficient functionality, results in low productivity and high process costs. The preliminary tests of the third concept, the dual 3D printing of single-layer sequins using PLA and ABS, shows that the printing process of the miniaturized functional sequins is successful. With a fine nozzle opening of the print head of approx. 0.4 mm, conductive structures of at least 0.4 mm width can be printed and embedded in a matrix made of conventional 3D printing material. In the third concept of dual 3D printing using conventional PLA material and a conductive filament, the conductive Electrifi filament requires a cost-intensive printing process due to an expensive market price of that filament. The standard print heads AA 0.4, AA 0.6 and BB 0.4 of the 3D printer Ultimaker 3 are not suitable for the conductive filament based on polyester and copper particles. The nozzles clog due to an insufficient nozzle opening and a narrow nozzle design. In order to counteract the clogging, an increased printing temperature for the conductive polyester filament is required, which leads to a blurring of extruded and non-solidified material through the nozzle during printing. This results in a non-uniformity of the fine conductive structures and the unevenness of printed surfaces with running contours. Areas from 1 cm² and lines with a width of at least 0.5 mm can be printed with the print heads AA 0.6 and BB 0.4. Considering the undertaken printing tests, functional 3D printed sequins cannot yet be produced productively with a conventional PLA filament and a conductive filament in this dual 3D printer.

### 3.3. Wearable Textile Application Using the Embroidery Process

**Figure 19:**
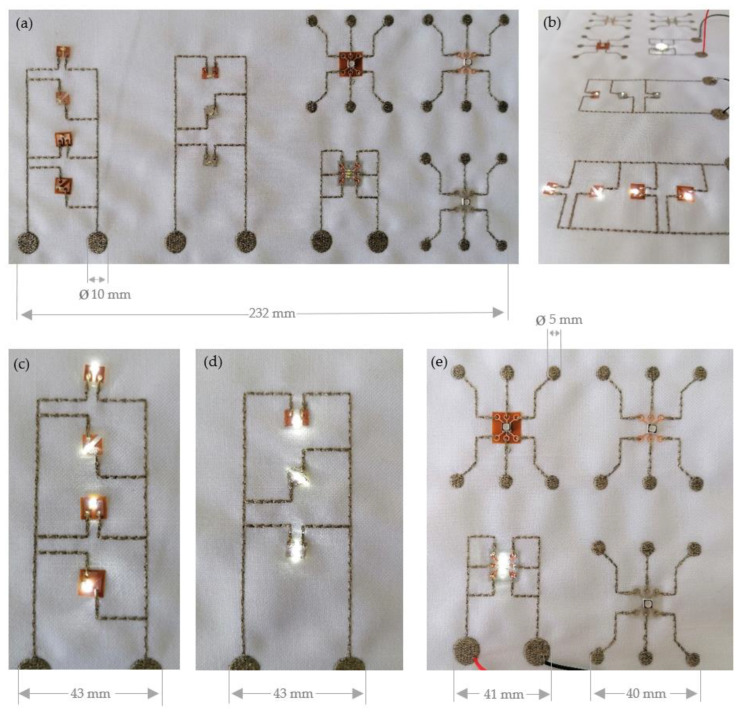
Embroidered circuit layouts: (**a**) Circuits for LED and RGB sequins without power source (**b**) overview of all Sequins with power source (**c**) LED sequins according to the subtractive technique (**d**) 3D printed LED sequins (**e**) RGB sequins by subtractive and additive technique.

To apply and contact the sequins, the embroidery design, respectively the electronic circuit for the functional sequins is first punched in the EPCwin embroidery software (ZSK Stickmaschinen GmbH, Krefeld, Germany). The electrically conductive data lines are embroidered automatically. The application of the sequins is partially automated by prelaying the sequins with subsequent contacting with a conductive embroidery thread (Figure 19).

The manufactured circuit pattern with flexible and functional sequins is reproducible and the duration of the embroidery process is short. That makes the process both effective and productive. The usability of the two functional sequins using subtractive and additive techniques is comparable and both variants can be applied and embroidered easily so that process-related no significant differences can be observed. From a mechanical point of view, the additively produced functional sequins are slightly thicker and less flexible, whereas the functional sequins produced with the subtractive technique are more flexible and adapt easily to the textile. Though thickness of sequins is regarded disadvantageous, the extending additively produced sequins do not influence the breakability of the conductive embroidery yarn, as the edges of the sequins are not sharp-edged but rounded. The light up of the LEDs serves as a functional test of the electronic properties, which is successfully passed for both manufactured sequin variants.

## 4. Conclusions

With the subtractive technology process of wax printing to mask a positive conductor pattern and a subsequent etching process to remove excess copper, one-sided sequins can produced as sequin tapes. The manufacturing of double-sided printed circuit boards is also possible. To develop the flexible and functional sequins, a PET film with copper adhesive tape and a copper-clad printed circuit board made of epoxy glass fiber laminate, are provided with a negative conductor pattern using wax printing with paraffin. Excess copper is etched away in a solution of sodium peroxodisulfate. Subsequent cleaning of the printed circuit boards is followed by assembly with the SMD components using a conductive adhesive with silver particles. The developed sequins of the PET film with copper adhesive tape offer uniform, sharply contoured conductive structures and a shiny and smooth copper and film surface. The fixation of the glued copper surfaces is not sufficiently adhesive in the outer adhesive edges and detaches under mechanical stress, e.g., slightly abrasion. The etching time of the etching process is very short, whereas removing the wax and the adhesive residues is very time-consuming and damages the conductor structures. The flexible and functional sequins made of copper-clad printed circuit boards have a matt and rough surface with slightly corrugated contours of the copper surfaces. The etching process takes a little longer than with the PET film. Post-processing by cleaning the copper surface or removing the wax is done quickly. In addition, no adhesive residues have to be removed, so that the contours of the copper surfaces are not attacked by the used alcohol. A conductive silver adhesive is used to equip the LED and RGB sequins tapes, which guarantees a high degree of flexibility of the contact points. Gluing is done manually; however, this process step can easily by automated using pick and place.

With the additive manufacturing respectively 3D printing, using fused filament fabrication (FFF), three different manufacturing concepts are processed. Based on the concept of 3D printing and gluing the conductor structures with copper adhesive tape, flexible and functional sequins with assembled LEDs and RGBs are produced as individual sequins and as a sequin tape. Cut edges for possible application and contact using embroidery technology can be integrated into the printed sequin tape. The printing time is short. Manual gluing of the conductive surfaces and structures proves to be time-consuming and unproductive. Moreover, it is not possible to automate the process step. Metallizing the conductive structures using gold sputtering after the dual 3D printing of a PVA masked on a PLA sequin does not lead to a conductive and uniform coating of the sequin surface. Sputtering leads to insufficient functionality of the sequins and low productivity due to high process costs. 3D printing of miniaturized sequins using the dual 3D printing principle has proven to be successful with conventional materials made of PLA and ABS. Fine structures with a line width of only 0.4 mm can be integrated into a carrier matrix and printed. However, using a special conductive filament made of polyester and copper particles, the printed functional sequins are less uniform and fine structures are not printable. To print the conductive filament, a special print head design with a very fine nozzle opening, which prevents the nozzles from clogging at the same time, is required. The existing standard print heads AA 0.4, AA 0.6 and BB 0.4 are not suitable for printing fine structures with the Electrifi filament and only allow the printing of large areas. The printing tests carried out for the production of 3D printed flexible and functional sequins using three different processes can currently not be assessed as suitable. 3D printing of dual miniaturized sequins is successful with conventional 3D printing materials and print heads, but cannot yet be transferred to the special conductive filaments on conventional 3D printers.

Further, more complex or multilayered structures or even printed circuit boards could be manufactured as functional sequins equipped with other components, e.g., multipolar electronic components. It is also conceivable to combine the flexible yet functional and textile-integrated electronic sequins with encapsulation techniques for wearable textiles. In addition, the functionality of the electronic components should further be characterized, such as the luminosity of the LEDs on different conductive structures of the functional sequins, as well as the specific material properties of the conductive structures.

## Figures and Tables

**Table 1:** 3D printing combination of materials and nozzles.

Print	Sequin Structure		Conductive Structure	
	Material	Nozzle	Material	Nozzle
1	PLA silver	AA 0.4	PLA black	AA 0.4
2	PLA transparent	AA 0.4	ABS gold	AA 0.4
3	PLA transparent	AA 0.4	ABS gold	AA 0.6

## Data Availability

The study did not report any data.
